# Molecular epidemiology of *bla*_*OXA-23*_-producing carbapenem-resistant *Acinetobacter baumannii* in a single institution over a 65-month period in north China

**DOI:** 10.1186/s12879-016-2110-1

**Published:** 2017-01-05

**Authors:** Nian-zhi Ning, Xiong Liu, Chun-mei Bao, Su-ming Chen, En-bo Cui, Ju-ling zhang, Jie Huang, Fang-hong Chen, Tao Li, Fen Qu, Hui Wang

**Affiliations:** 1 State Key Laboratory of Pathogen and Biosecurity, Beijing Institute of Microbiology and Epidemiology, No.20 Dongda Street, Fengtai District, 100071 Beijing, China; 2 The Center of Clinical Diagnosis Laboratory, Beijing 302 Hospital, Beijing, 100039 China

**Keywords:** *Acinetobacter baumannii*, Carbapenem resistant, *bla*_*OXA-23*_, CC92, Outbreak

## Abstract

**Background:**

Carbapenem-resistant *Acinetobacter baumannii* poses a significant threat to hospitalized patients, as few therapeutic options remain. Thus, we investigated the molecular epidemiology and mechanism of resistance of carbapenem-resistant *A.baumannii* isolates in Beijing, China.

**Methods:**

Carbapenem-resistant *A.baumannii* isolates (*n* = 101) obtained between June 2009 and November 2014 were used. Multilocus sequence typing (MLST) and PCR assays for class C and D β-lactamase were performed on all isolates. S1 nuclease pulsed-field gel electrophoresis (PFGE) and Southern blot hybridization were performed to identify the resistance gene location.

**Results:**

All 101 *A.baumannii* isolates were highly resistant to frequently used antimicrobials, and were considered multidrug resistant. A total of 12 sequence types (STs) were identified, including 10 reported STs and 2 novel STs. Eighty-seven isolates were classified to clonal complex 92 (CC92), among which ST191 and ST195 were the most common STs. The *bla*_*OXA-23*_ gene was positive in most (*n* = 95) of the *A.baumannii* isolates. Using S1-nuclease digestion PFGE and Southern blot hybridization, 3 patterns of plasmids carrying *bla*_*OXA-23*_ were confirmed. ST191 and ST195 (both harboring *bla*_*OXA-23*_) caused outbreaks during the study period, and this is the first report of outbreaks caused by ST191 and ST195 in north China.

**Conclusion:**

*bla*_*OXA-23*_-producing *A.baumannii* ST191 and ST 195 isolates can disseminate in a hospital and are potential nosocomial outbreak strains. Surveillance of imipenem-resistant *A.baumannii* and antimicrobial stewardship should be strengthened.

## Background

*Acinetobacter baumannii* is an opportunistic pathogen involved in outbreaks occurring in burn units, surgical wards or intensive care units (ICUs), as well as important cause of nosocomial septicemia, pneumonia and urinary tract infections [1]. *A.baumannii* is of interest due to increasing the increase in antimicrobial resistance [2]. This organism is generally intrinsically resistant to many frequently-used antibiotics, including aminopenicillin, first- and second-generation cephalosporins and chloramphenicol [3, 4]. Carbapenems are important antibiotics to treat *A.baumannii* because they are highly efficacious and have low toxicity [5]. However, the emergent and rapid spread of carbapenem-resistant *A.baumannii* isolates pose a severe threat to public health and are a global concern [6]. Carbapenem resistance, such as to imipenem, increased in China from 31.0% in 2005 to 62.4% in 2014 [7]. Recent studies also suggest high resistant of *A.baumannii* against carbapenems across the world [8–11].

Carbapenem resistance in *A.baumannii* is mainly mediated by the production of carbapenem-hydrolyzing enzymes [6]. Class D OXA-type enzymes are the most prevalent carbapenemases in *A.baumannii* [12]. In addition to the intrinsic OXA-51-like enzymes, 3 unrelated groups of these carbapenem-hydrolysing enzymes have been identified OXA-23-like, -40-like and -58-like [13]. Outbreaks of *bla*_*OXA-23*_-producing *A.baumannii* have been reported across the world [14–16] and a previously study has pointed out that *bla*_*OXA-23*_ was the predominant group of carbapenem-hydrolysing enzymes in China [17].

Multilocus sequence typing (MLST) is used for global and long-term epidemiological studies [18], and data from MLST show that CC92 was the most widely distributed *A.baumannii* clone globally [19–21]. Studies from China indicate that *bla*_*OXA-23*_-producing CC92clones are prevalent in most provinces of China [17, 22]. Although the molecular epidemiology of carbapenem-resistant *A.baumannii* has been investigated, the epidemiology of carbapenem-resistant *A.baumannii* over long time periods in single institution may allow new insights into the behavior of this pathogen.

Thus, we sought to investigate carbapenem-resistance mechanisms and the molecular epidemiology of carbapenem-resistant *A. baumannii* in a single hospital over a 65-month period.

## Methods

### Bacterial isolates

Between June 2009 and November 2014, a total of 101 nonduplicate carbapenem-resistant (Zone Diameter of imipenem ≤18 mm; Clinical Laboratory Standards Institute [CLSI] breakpoint) *A. baumannii* (CRAB) isolates were collected from a single hospital in Beijing, China. A single isolate per patient was included. All isolates were identified by conventional biochemical techniques using VITEK 2 system (BioMérieux France). PCR confirmation of the *bla*_*OXA-51-like*_ carbapenemase gene was performed to help identify *A. baumannii* simultaneously, because this gene is intrinsic to *A. baumannii* [23, 24].

### Antimicrobial susceptibility testing

The disk diffusion method was used to evaluate susceptibility to the following antimicrobial agents: imipenem (IPM: 10 μg), ceftazidime (CAZ: 30 μg), amikacin (AMK: 30 μg), piperacillin/tazobactam (TZP: 100/10 μg), levofloxacin (LVX: 5 μg), ticarcillin/Clavulanic acid (TCC: 75/10 μg), minocycline (MNO: 30 μg) (Oxoid, UK). Results were interpreted in accordance with CLSI guidelines from 2011. Isolates with intermediate susceptibility were classified as non-susceptible.

### Molecular typing methods

Multilocus sequence typing (MLST) was performed on all *A.baumannii* isolates as described previously [18]. Analysis of allele sequences and sequence type (ST) assignment made use of the Oxford *Acinetobacter baumannii* MLST website (http://pubmlst.org/abaumannii/). The eBURST algorithm (version 3; http://eburst.mlst.net/) was used to assign clonal complexes (CCs).

### Screening of ambler classes C and D β-lactamase genes

PCR experiments were carried out using primers specific for the genes encoding Ambler C and D β-lactamase (AmpC, MOX-1, MOX-2, CMY-1 to CMY-11, BIL-1, DHA-1, DHA-2, ACC, ACT-1, MIR-1 T, FOX-1 to FOX-5b, bla_OXA-23-like_, bla_OXA-40-like_, bla_OXA-51-like_, and bla_OXA-58-like_ and bla_OXA-143_)as described previously [25–28]. Primers are depicted in Table 1. For each gene detected, some PCR products were randomly selected, and then sequenced to confirm genes.

**Table 1:** Primers used in this study

Primer	Sequence(5′ to 3′)	Target	Reference
MOXMF	GCTGCTCAAGGAGCACAGGAT	MOX-1, MOX-2, CMY-1, CMY-8 to CMY-11;	[25]
MOXMR	CACATTGACATAGGTGTGGTGC		
CITMF	TGGCCAGAACTGACAGGCAAA	LAT-1 to LAT-4, CMY-2 to CMY-7, BIL-1;	[25]
CITMR	TTTCTCCTGAACGTGGCTGGC		
DHAMF	AACTTTCACAGGTGTGCTGGGT	DHA-1, DHA-2	[25]
DHAMR	CCGTACGCATACTGGCTTTGC		
ACCMF	AACAGCCTCAGCAGCCGGTTA	ACC	[25]
ACCMR	TTCGCCGCAATCATCCCTAGC		
EBCMF	TCGGTAAAGCCGATGTTGCGG	MIR-1 T ACT-1	[25]
EBCMR	CTTCCACTGCGGCTGCCAGTT		
FOXMF	AACATGGGGTATCAGGGAGATG	FOX-1 to FOX-5b	[25]
FOXMR	CAAAGCGCGTAACCGGATTGG		
AmpCF	ACAGAGGAGCTAATCATGCG	AmpC	[26]
AmpCR	GTTCTTTTAAACCATATACC		
OXA-23-likeF	GATCGGATTGGAGAACCAGA'	bla_OXA-23-like_	[27]
OXA-23-likeR	ATTTCTGACCGCATTTCCAT		
OXA-40-likeF	GGTTAGTTGGCCCCCTTAAA	bla_OXA-40-like_	[27]
OXA-40-likeR	AGTTGAGCGAAAAGGGGATT		
OXA-51-likeF	TAATGCTTTGATCGGCCTTG	bla_OXA-51-like_	[27]
OXA-51-likeR	TGGATTGCACTTCATCTTGG		
OXA-58-likeF	AAGTATTGGGGCTTGTGCTG	bla_OXA-58-like_	[27]
OXA-58-likeR	CCCCTCTGCGCTCTACATAC		
OXA-143	TGGCACTTTCAGCAGTTCCT	bla_OXA-143_	[28]
OXA-143	TAATCTTGAGGGGGCCAACC		

### PFGE and Southern blot hybridization

To detect plasmids of A.baumannii isolates, an agarose gel plug containing total cellular DNA was prepared and digested with S1 nuclease (Takara, Japan) as described previously [29]. Digested plugs were subjected to PFGE using a CHEF-Mapper system (pulse times, 5 to 30 s; running time, 15 h; 6 V/cm). Gels were blotted onto nylon membranes (Millipore, USA) using standard techniques. The membrane was hybridized with a digoxigenin-labeled probe consisting of a *bla*_*OXA-23*_ fragment which was amplified by primers.

## Results

A total of 101 *A.baumannii* isolates were resistant to imipenem and considered carbapenem-resistant and enrolled in our study. CRAB isolates were obtained from various sources, including sputum (*n* = 72 isolates), blood (*n* = 14 isolates), abdominal fluid (*n* = 9 isolates), secretion (*n* = 2 isolates), catheter (*n* = 1 isolates), eyes (*n* = 1 isolates), pus (*n* = 1 isolates) and throat swabs (*n* = 1 isolates). Of 101 CRAB isolates, 87% (*n* = 88) were collected from the ICU. The temporal distribution of CRAB isolates is showed as follows. 2, 3, 45, 4, 18 and 29 isolates were obtained in 2009, 2010, 2011, 2012, 2013 and 2014, respectively. CRAB isolates resistance data appear in Table 2. All CRAB isolates were resistant to at least 3 classes of antibiotic and were considered multidrug resistant. CRAB isolate data for AmpC and *bla*_O*XA-51-like*_ genes appear in Table 2.

**Table 2:** Details of *A. baumannii* isolates, by sequence type

				Non-susceptible to (%):							Resistant determinants
ST	No.	Allelic profile	Time course	IPM	TZP	TCC	CAZ	AMK	LVX	MNO	
191	32	1-3-3-2-2-94-3	Aug. 2009 Feb. 2011–May. 2012	100	100	100	100	38	100	23	OXA-51, OXA-23, AMPC
195	31	1-3-3-2-2-96-3	Mar. 2013–Nov. 2014	100	100	100	100	97	100	100	OXA-51, OXA-23, AMPC
208	15	1-3-3-2-2-97-3	Jul. 2010–Oct. 2014	100	100	100	93	100	100	100	OXA-51, OXA-23, AMPC
218	2	1-3-3-2-2-102-3	Jul. 2011–Aug. 2011	100	100	100	100	100	100	0	OXA-51, OXA-23, AmpC
368	6	1-3-3-2-2-140-3	Jun. 2009–Sep. 2014	100	100	100	100	67	67	33	OXA-51, OXA-23, AmpC
369	1	1-3-3-2-2-106-3	Jun. 2013	100	100	100	100	0	100	-	OXA-51, OXA-23, AmpC
373	2	1-12-12-11-4-103-3	Mar. 2011, Apr. 2011	100	100	100	100	0	100	0	OXA-51, AmpC
383	2	1-12-56-1-4-149-45	Aug. 2011	100	100	100	100	100	100	100	OXA-51, AmpC
429	2	1-34-56-1-4-144-45	Jul. 2011, Sep. 2011	100	100	100	100	50	50	100	OXA-51, OXA-23, AmpC
469	6	1-12-3-2-2-103-3	Dec. 2012-Nov. 2013	100	100	100	100	100	100	100	OXA-51, OXA-23, AmpC
1302^n^	1	2-52-80-6-23-140-4	Jul. 2014	100	100	100	0	0	0	0	OXA-51, OXA-40, AmpC
1309^n^	1	27-155^n^-99-55-25 -270^n^-60	Aug. 2011	100	100	100	100	100	100	100	OXA-51, AmpC
Total	101	-	Jun. 2009–Nov. 2014	100	100	100	98	80	96	68	-

^n^Novel; *IPM* imipenem, *TZP* piperacillin/tazobactam, *TCC* ticarcillin/clavulanic acid, *CAZ* ceftazidime, *AMK* amikacin, *LVX* levofloxacin, *MNO* minocycline

**Fig. 1:**
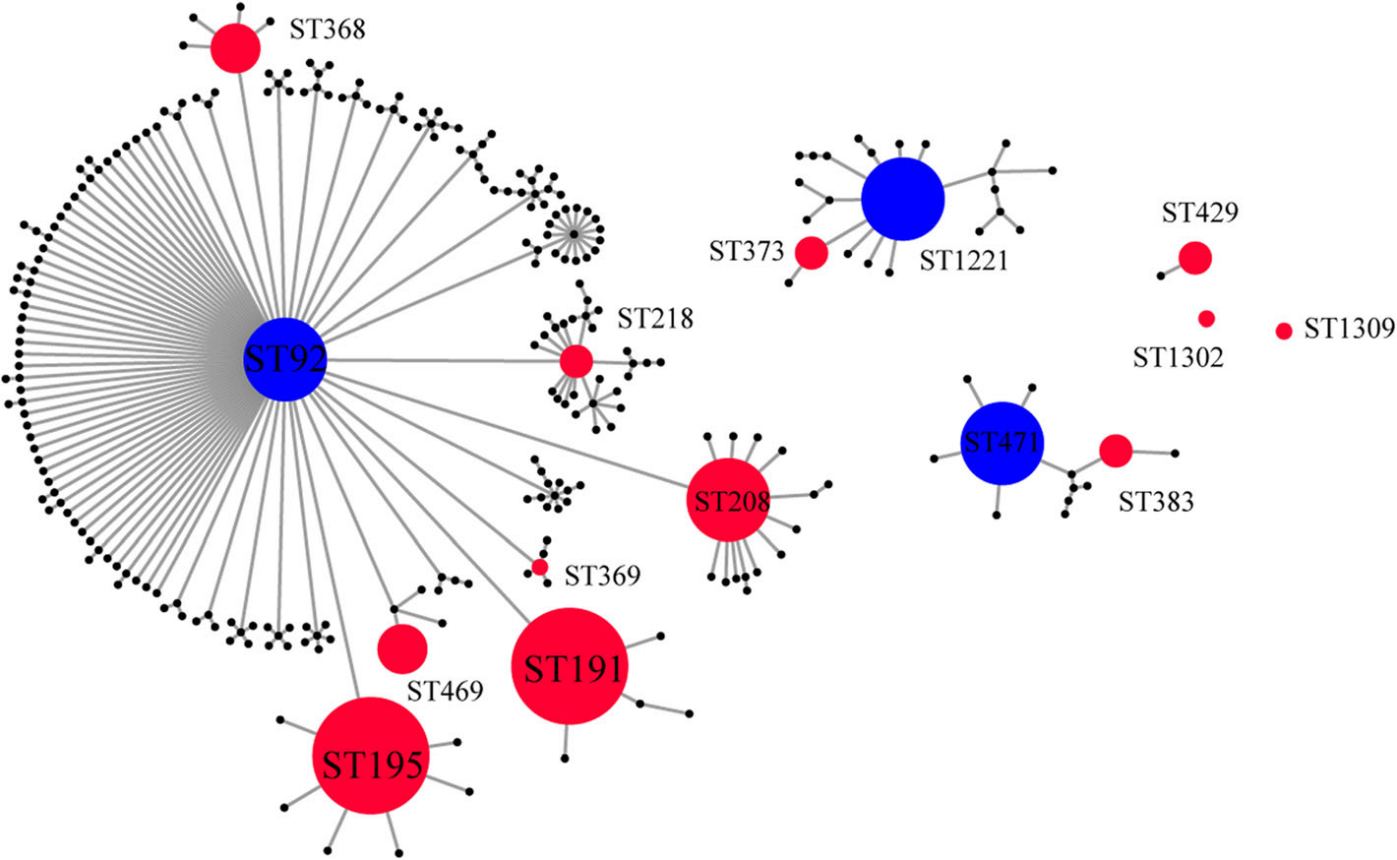
eBURST results of 12 STs presented in this study. The 1309 STs in the entire *A.baumannii* MLST database were analyzed using the most stringent definition (6/7 shared alleles). Four groups and 2 singletons that included STs found in our study are displayed as an eBURST diagram together. Each circle represents an ST. STs in a group are considered to belong to same clonal complex. Blue STs are founders of corresponding group. Red STs were found in this study. Circle size reflects the number of strains. Other ST labels have been removed for clarity

To investigate the molecular epidemiology of isolates, MLST was performed to characterize CRABs and data are summarized in Table 2. The eBURST analysis data appear in Fig. 1.

**Fig. 2:**
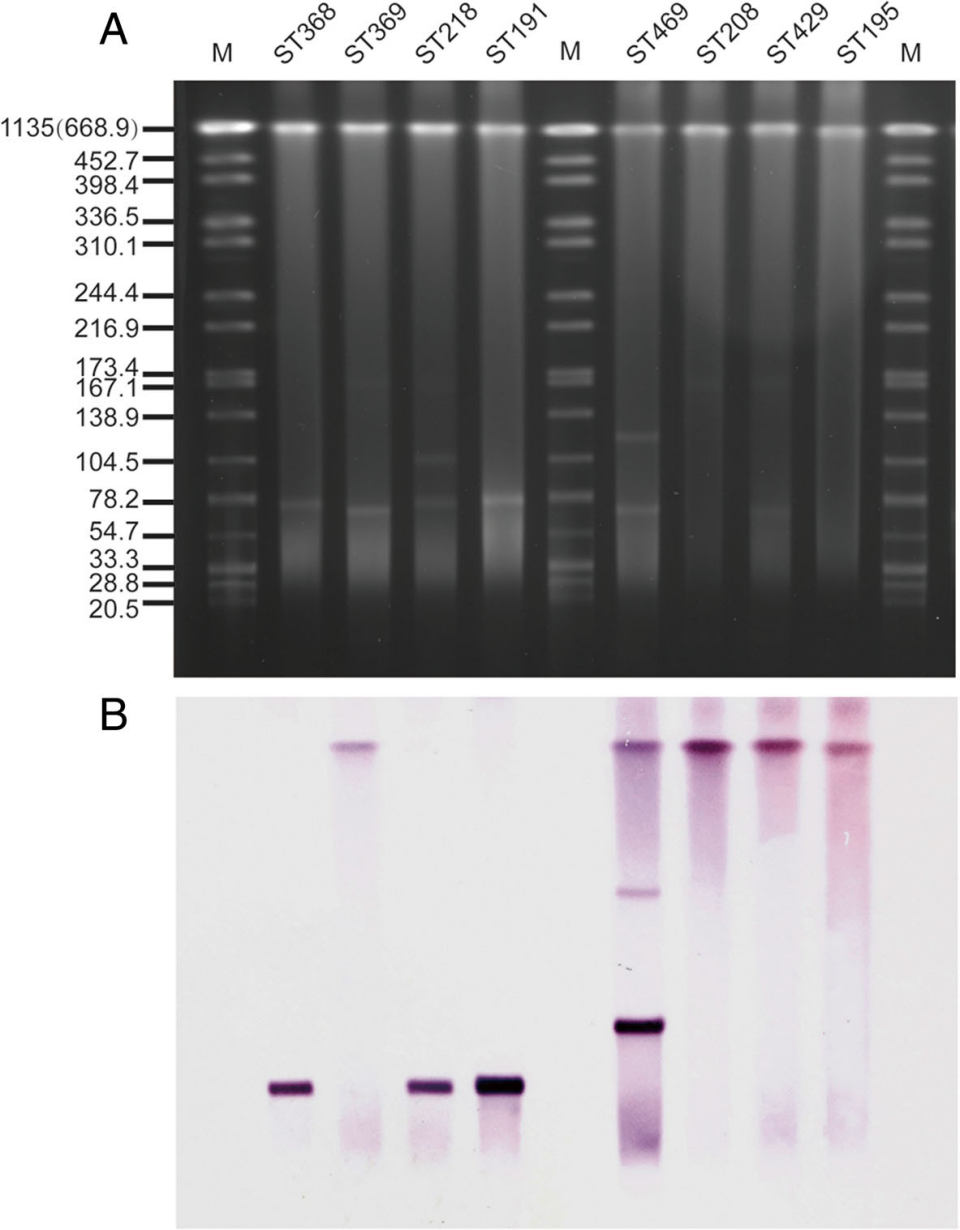
Results of plasmid measurement and hybridization. **a** S1 nulcease digestion of total DNA of A.baumannii isolates was followed by PFGE. Plasmid bands are shown as linearized fragments on the gel. For each A.baumannii ST, only one strain was selected randomly for this assay. **b** Southern blot hybridization for the *bla*_*oxa-23*_ gene. Lane M, reference standard strain H9812 restricted with XbaI. MW are in KB

ST191 was the largest sequence type (32 of 101 isolates) and was found in our institution from August 2009 to May 2012. Only 1 isolate was obtained in August 2009 from the Liver Failure ward, but most (96.9%) of ST191 isolates were collected between February 2011 and May 2012. Importantly, 28 of the 32 ST191 strains were isolated from ICU ward. All ST191 isolates were resistant to piperacillin/tazobactam, ticarcillin/clavulanic acid, ceftazidime and levofloxacin but had variable susceptibilities to amikacin and minocycline (Table 2). All of ST191 isolates were *bla*_*OXA-23*_-positive. One strain was selected randomly to be subjected to PFGE digested with S1 nuclease, and results show that this ST strain contains a plasmid of approximately 78 Kb. Southern blot hybrid hybridization assays confirmed that *bla*_*OXA-23*_ gene was located on this plasmid (Fig. 2).

Thirty-one (30.7%) isolates were defined as ST195, and this ST was first detected in the infectious liver diseases ward in March 2013. Twenty-eight ST195 isolates were collected from ICU ward from then on. It is notable that all ST195 isolates were resistant to almost all antibiotics tested in this study except 1 isolate was susceptible to amikacin. All of ST195 isolates were also *bla*_*OXA-23*_ positive. This ST strain does not contain any plasmid, and the *bla*_*OXA-23*_ gene is located on the chromosome (Fig. 2).

Another 4 sequence types, ST208, ST368, ST218 and ST369, were found in 15 (14.8%), 6 (5.9%), 2 (2.0%) and 1 (1.0%) isolates, respectively, and carried the *bla*_*OXA−23*_ gene but had different resistance profiles. The *bla*_*OXA−23*_ gene is located on the plasmid in the *A.baumannii* ST218 and ST368 strain, but is found on the chromosome in ST208 and ST369.

Six isolates were ST469. This is a double-locus variant (DLV) of multiple STs within CC92, implying a close relationship. However, it does not agree with the conservative definition of sharing alleles at 6/7 of the loci, and thus ST469 cannot be considered a CC92 member. All ST469 isolates were collected during December 2012 and November 2013. ST469 isolates were *bla*_*OXA-23*_ positive and were resistant to allantibiotics. PFGE and hybridization results show that this ST isolate harbored 2 plasmids. The *bla*_*OXA-23*_ gene was located on a ca. 120 kb plasmid and on a plasmid of approximately 245 kb, with another copy of *bla*_*OXA-23*_ on a chromosome (Fig. 2).

Two unreported singleton STs were identified. ST1309 presented in this hospital in August 2011 only and was resistant to all the 7 classes of antimicrobials. The carbapenem-resistant determinant of this isolate remains unclear. The ST1302 isolate was unique among imipenem-resistant strains; it was ceftazidime, amikacin, levofloxacin and minocycline susceptible and was the only isolate that carried the *bla*_*OXA-40*_ gene.

## Discussion

This study offers insight into the longitudinal evaluation of the molecular epidemiology of carbapenem-resistant *A.baumannii* in a single institution over a 65-month period.

The *bla*_*OXA-23*_ gene was positive in most (*n* = 95) of the *A.baumannii* isolates in this institution. The first report of this enzyme in *A.baumannii* was ARI-1, which was identified in an isolate from Scotland collected in 1985 [30]. In 2000, enzyme sequence analysis (re-named OXA-23) indicated that it was a member of the ambler class D group of β-lactamases [31]. Since then, outbreaks of OXA-23 carbapenemase-producing A.baumannii have been reported all over the world [32–36]. Our finding was consistent with other reports from China. Zhou’s group investigated resistance determinants of 342 imipenem-resistant *A.baumannii* isolates which were collected from 16 Chinese cities in 2005, and found that most CRAB isolates contained the *bla*_*OXA-23*_ gene [37]. Recent studies confirm a high prevalence of the *bla*_*OXA-23*_ gene in carbapenem-resistant *A.baumannii* in different Chinese cities (80.6-100%) [38–40]. Southern blotting revealed that the *bla*_*oxa-23*_ gene is plasmid-mediated in some STs (ST191, ST218 and ST368), but chromosome borne in others (ST195, ST208, ST369 and ST429). Chromosomal locations of *bla*_*oxa-23*_ make it less likely for *A.baumannii* to lose caarbapenem resistance. Investigation of OXA-23 producing *A.baumannii* isolates collected from 28 hospitals in 18 provinces of China showed that OXA-23 was mainly located on a ca.78-kb plasmid or on a chromosome [41].

We were concerned that *A.baumanii* isolates harboring the *bla*_*OXA-23*_ gene were multidrug resistant in our study and had few antibiotic therapeutic options for treating CRAB infection*.* Thus, controlling the spread of *bla*_*OXA-23*_ producing *A.baumannii* is important.

We identified ST191, ST195, ST208, ST218, ST368 and ST369 as classified into CC92 which was the largest and most widely distributed *A.baumannii* clone in China [17]. CC92 represented the most epidemic CRAB STs in this hospital, accounting for 86.1% of isolates in this study. For *bla*_*OXA-23*_-producing CRAB of CC92, the ability to disseminate in a single institution for a long time suggests that adaptation to the hospital environment may be important for the success of *A.baumannii*.

It has been suggested that any clinical *A.baumannii* isolates with resistance to multiple antibiotics can cause a nosocomial outbreak [42]. We found that imipenem-resistant *A.baumannii* of CC92, compared with other clonal complexes, may be more prone to cause severe outbreaks during long-term dissemination. Two outbreaks of CRAB CC92 were observed in our institution. Most ST191 isolates (31/32) were identified between February 2011 and May 2012 in the ICU ward, suggesting an outbreak of *bla*_*OXA-23*_-producing ST191. Deng’s group has reported the prevalence of an *A.baumannii* ST191 clone in a southern Chinese hospital [43]. To our knowledge, this is the first identification of an outbreak of *bla*_*OXA-23*_ harboring *A.baumannii* ST191 isolate in north China. A second outbreak of *bla*_*OXA-23*_-producing CRAB occurred in this ICU later. Thirty-one *bla*_*OXA-23*_-producing ST195 isolates were also found in the ICU between March 2013 and November 2014and this sequence type was more resistant to frequently-used antimicrobial agents compared with ST191. ST195 has frequently been identified in Asian countries, including Japan, Vietnam, and Malaysia [44–46]. To our knowledge, Li’s group was first to identify ST195 in a teaching hospital in Guangzhou, in southern China [47]. Since then, ST195 clones has been identified in western and eastern China [38, 48]. Here, we offer the first report of an outbreak of *bla*_*OXA-23*_-producing ST195 in north China, suggesting that ST195 has been successfully disseminated in this country.

We collected no environmental strains from the work place, so we lack surveillance for source identification, which is a significant limitation of our study.

## Conclusions

In summary, *bla*_*OXA-23*_-producing CC92 isolates were prevalent in this hospital over a 65-month period. Successive outbreaks of ST191 and ST195 demonstrated that persisting clinical carbapenem-resistant *A.baumannii* isolate can cause a nosocomial outbreak. Periodic investigation of molecular epidemiology and resistance determinant of *A.baumannii* is necessary.

## Abbreviations

CC: Clonal complex 92
CRAB: Carbapenem-resistant *A. baumannii*
DLVs: Double-locus variants
MLST: Multilocus sequence typing
PFGE: Pulsed-field gel electrophoresis
SLVs: Single-locus variants
ST: Sequence type
